# Invasive Pseudomembranous *Aspergillus* Tracheobronchitis Accompanied With Fever and Hemoptysis: A Case Report

**DOI:** 10.1002/ccr3.70805

**Published:** 2025-08-25

**Authors:** Xiaolong Li, Xin Wang, Shuhao Xu, Yinhe Feng, Yuanyuan Liu, Chunfang Zeng

**Affiliations:** ^1^ Pulmonary and Critical Care Medicine People's Hospital of Deyang City Deyang Sichuan China; ^2^ Department of Stomatology People's Hospital of Deyang City Deyang Sichuan China

**Keywords:** bronchoscopy, diagnosis, pseudomembranous *Aspergillus* tracheobronchitis, treatment

## Abstract

Pseudomembranous *Aspergillus* tracheobronchitis is a relatively rare disease, and it is very easy for clinicians to misdiagnose. It is particularly necessary for the patient presenting with recurrent pyrexia and hemoptysis in the absence of clear‐cut pulmonary radiological findings to receive a bronchoscopy.

**Abbreviations**
AIDS: acquired immune deficiency syndrome
COPD: chronic obstructive pulmonary disease
HbA1c: glycosylated hemoglobin
HIF‐1α: hypoxia‐inducible factor‐1α
IPA: invasive pulmonary aspergillosis
NOX: NADP oxidase
ROS: reactive oxygen species

## 1 Introduction

Invasive *Aspergillus* tracheobronchitis represents an uncommon manifestation of *Aspergillus* infection, localized predominantly to the tracheobronchial tree with significant diagnostic and therapeutic challenges [1]. Unlike classical invasive pulmonary aspergillosis (IPA), which primarily affects the lung parenchyma in profoundly immunocompromised hosts, invasive *Aspergillus* tracheobronchitis—particularly its pseudomembranous variant—occurs sporadically and often evades timely diagnosis due to nonspecific symptoms and absence of characteristic radiological findings. This entity manifests histopathologically as fungal hyphal invasion beneath a necroinflammatory pseudomembrane, frequently complicating conditions, such as prolonged corticosteroid use, hematologic malignancies, or solid organ transplantation [2, 3]. Notably, emerging evidence suggests invasive *Aspergillus* tracheobronchitis may also afflict patients with milder immune perturbations, including chronic obstructive pulmonary disease (COPD), diabetes mellitus, or viral respiratory infections [4]. Despite advances in antifungal therapies, pseudomembranous tracheobronchitis remains prone to misdiagnosis as endobronchial tuberculosis or bacterial bronchitis, often delaying appropriate intervention. Bronchoscopy with histopathological confirmation thus constitutes a critical diagnostic tool in such scenarios. Herein, we present a case of pseudomembranous *Aspergillus* tracheobronchitis in a diabetic COPD patient with influenza coinfection, highlighting the pivotal role of bronchoscopic evaluation in atypical presentations of recurrent fever and hemoptysis [5].

## 2 Case Presentation

### 2.1 Case History

A 63‐year‐old male was admitted to the hospital after experiencing a week of recurrent pyrexia and minimal hemoptysis. The patient had a documented history of chronic obstructive pulmonary disease and was receiving long‐term treatment with fluticasone furoate/umeclidinium/vilanterol. The patient has previously suffered from type 2 diabetes and has long‐term oral administration of metformin tablets (500 mg twice daily). However, glycemic control remained suboptimal during the week preceding admission, with blood glucose levels fluctuating between 9.1 and 22.6 mmol/L.

### 2.2 Differential Diagnosis, Investigations, and Treatment

**FIGURE 1:**
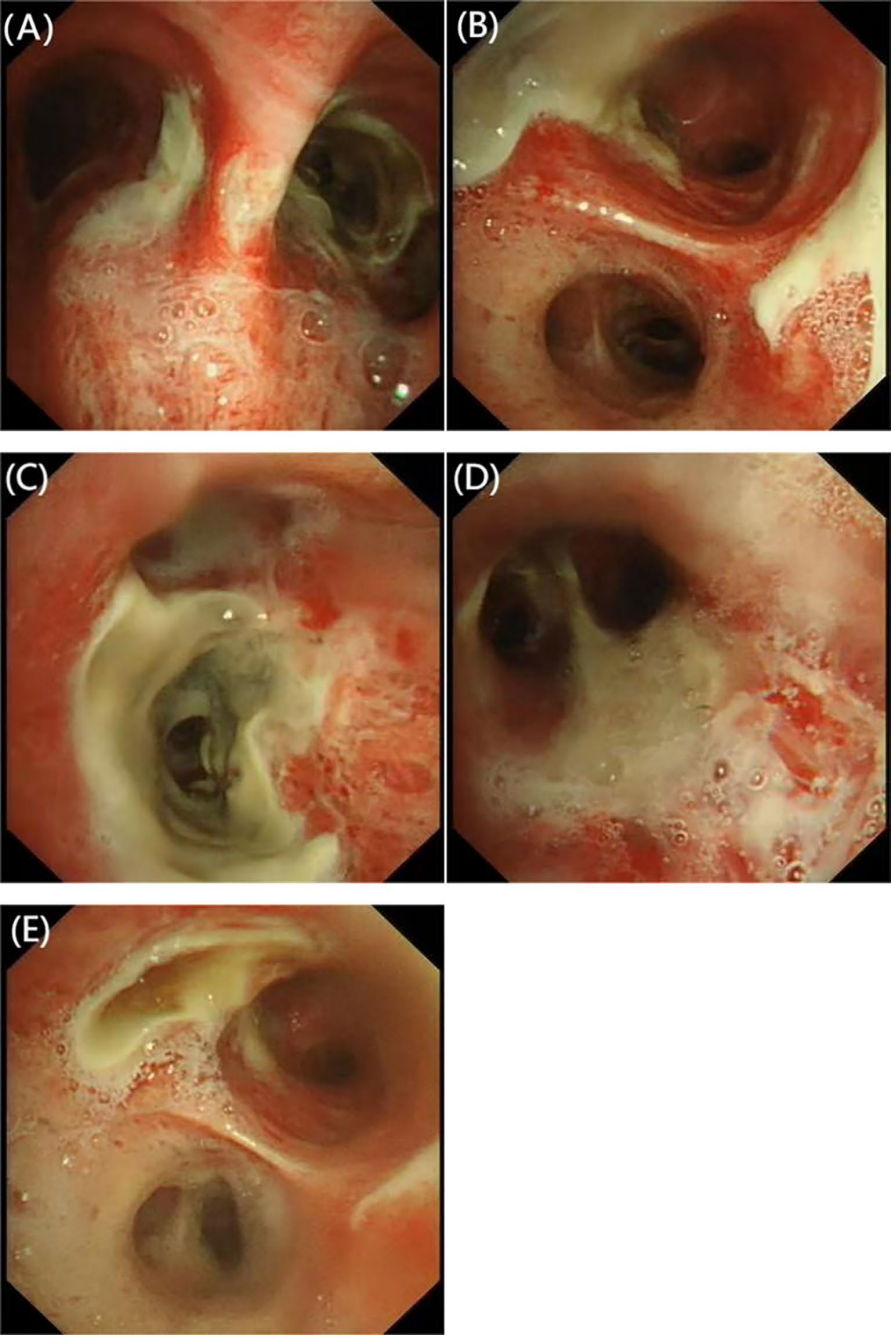
(A–E) Yellow–white pseudomembrane on the tracheal and bronchial surfaces, friable and bled upon contact.

**FIGURE 2:**
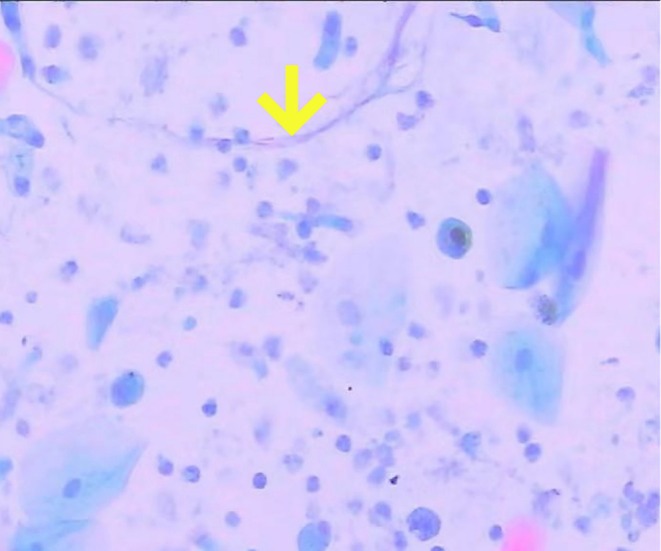
Fungal spores and *Aspergillus* hyphae were identified in lavage fluid by high magnification (H&E, ×20 magnification).

**FIGURE 3:**
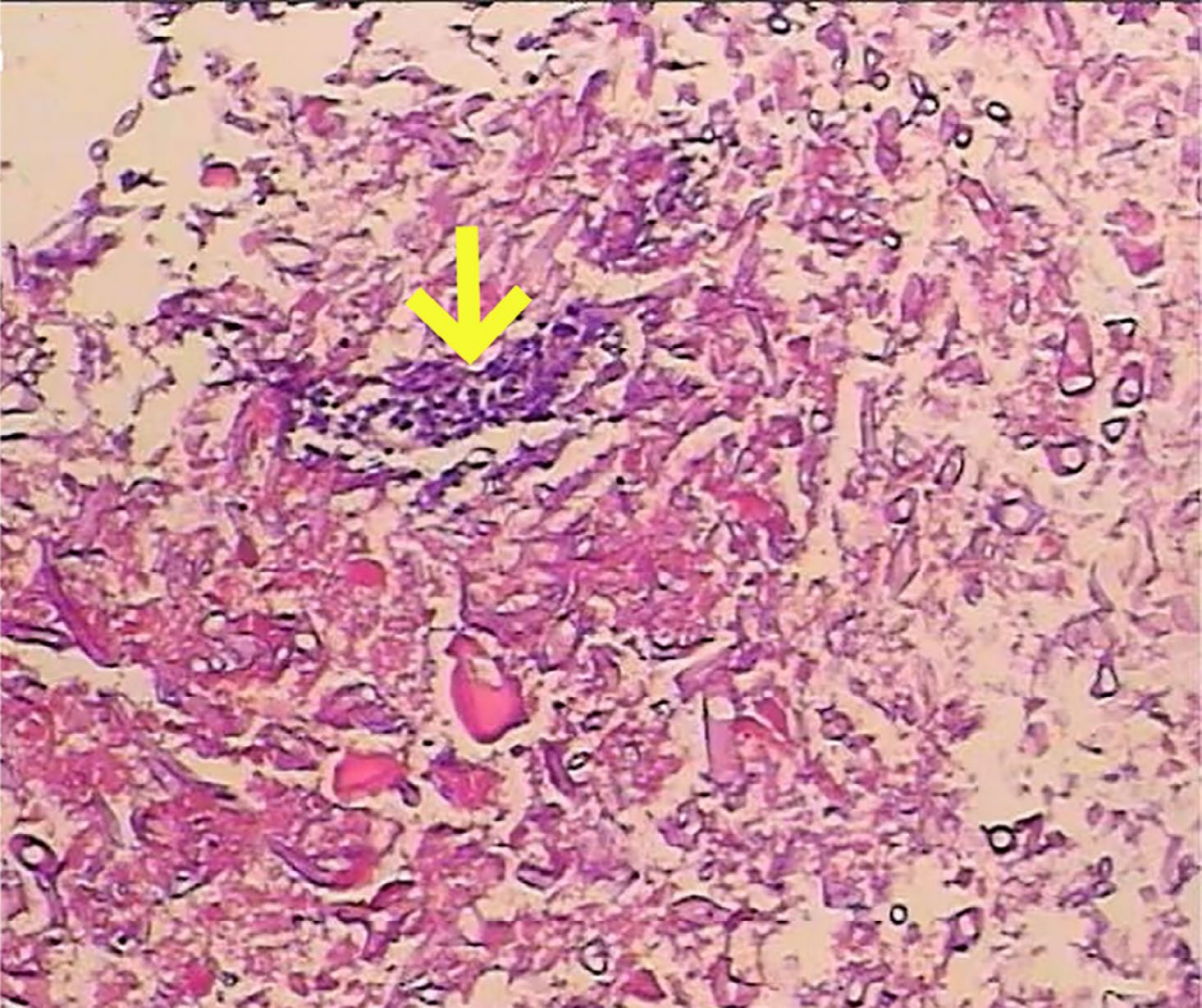
Histopathological analysis of the tracheal mucosal biopsy specimen demonstrated evidence of *Aspergillus* hyphae (H&E, ×20 magnification).

Upon admission, laboratory tests revealed leukocytosis with a count of 12.4 × 10^9^/L, neutrophilia at 9.6 × 10^9^/L, and a hemoglobin level of 117 g/L. The highly sensitive C‐reactive protein level was notably elevated at 182 mg/L. Thoracic computed tomography showed findings consistent with pulmonary emphysema and bullae. Additionally, the patient displayed febrile symptoms, and a pharyngeal swab tested positive for influenza A nucleic acids. Upon hospital admission, the patient's blood glucose was 21.9 mmol/L. Subsequent in‐hospital monitoring revealed fluctuations ranging from 10.2 to 25.8 mmol/L. The patient's glycosylated hemoglobin (HbA1c) value is 9.8%. Consequently, oral metformin therapy was discontinued, and intensified subcutaneous insulin therapy was initiated with the following regimen: regular human insulin (4 units before breakfast, 6 units before lunch, 6 units before dinner) and neutral protamine Hagedorn insulin (6 units at bedtime). The initial diagnoses included an acute exacerbation of COPD and influenza A infection. Despite the administration of intravenous ceftriaxone (2000 mg once daily for 6 days) for the patient's continued pyrexia and production of yellow, purulent sputum, along with the initiation of antiviral therapy using oseltamivir phosphate capsules (75 mg twice daily for 5 days) and hemostatic intervention with intravenous tranexamic acid (1000 mg once daily for 5 days), the patient continued to experience persistent minor hemoptysis. Subsequently, bronchoscopy was performed, revealing the presence of a yellow–white pseudomembrane on the tracheal and bronchial surfaces, which appeared friable and bled upon contact (Figure 1A–E). Tracheal lavage and mucosal biopsy of the tracheal lesions were then carried out. Examination of the lavage fluid at high magnification identified fungal spores and *Aspergillus* hyphae (Figure 2). Furthermore, histopathological analysis of the tracheal mucosal biopsy confirmed the presence of *Aspergillus* hyphae (Figure 3). The patient was finally diagnosed with pseudomembranous *Aspergillus* tracheobronchitis. During the hospitalization, intravenous voriconazole therapy was initiated with a loading dose of 6 mg/kg every 12 h on day 1, followed by a maintenance dose of 4 mg/kg twice daily. Concomitantly, inhaled voriconazole (40 mg once daily for 7 days) was administered. Following treatment, the patient became afebrile and hemoptysis markedly reduced.

## 3 Outcome and Follow‐Up

**FIGURE 4:**
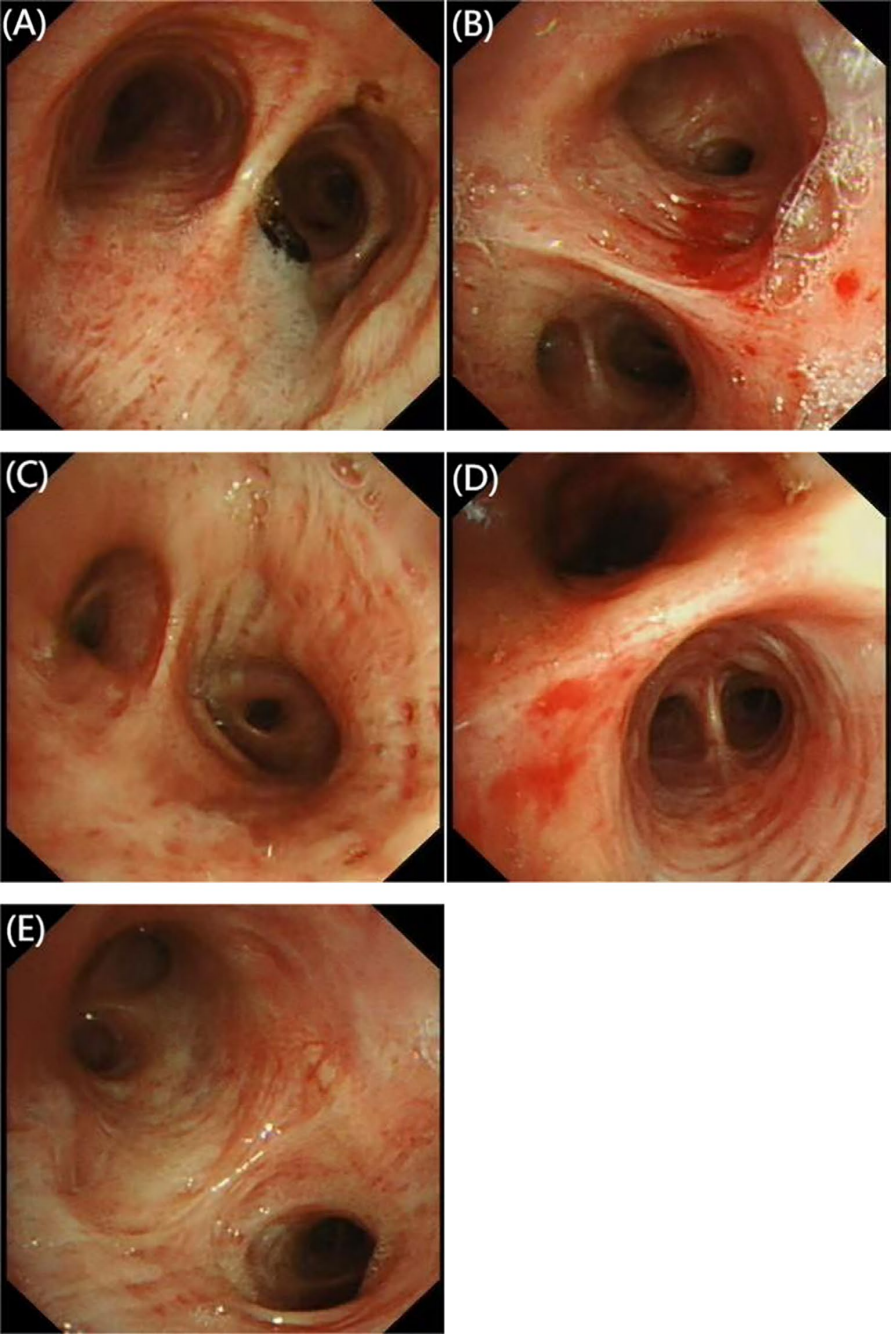
(A–E) A follow‐up bronchoscopy after 2 months showed significant recovery of the tracheal and bronchial mucosa.

Post‐discharge, the patient continued oral voriconazole (200 mg twice daily for 12 weeks). A follow‐up bronchoscopy after 2 months showed significant recovery of the tracheal and bronchial mucosa (Figure 4A–E).

## 4 Discussion

Invasive pulmonary aspergillosis stands out as the most prevalent fungal infection affecting the lungs in immunocompromised individuals, typically centered on the lung parenchyma, while tracheal and bronchial involvement remains an infrequent occurrence. Specifically, *Aspergillus* tracheobronchitis denotes an *Aspergillus* infection localized within the trachea and bronchi, presenting as a rare subset of invasive pulmonary aspergillosis [6].

In this case, the patient was diagnosed with pseudomembranous *Aspergillus* tracheobronchitis mainly relying on the presence of *Aspergillus* hyphae by mucosal biopsy and yellow–white pseudomembrane on the tracheal and bronchial surfaces by bronchoscopy. Pseudomembranous *Aspergillus* tracheobronchitis is a type of tracheobronchial aspergillosis. Pseudomembranous *Aspergillus* tracheobronchitis is relatively rare, which makes it easy for doctors to misdiagnose as endobronchial tuberculosis. Therefore, we wish to raise clinicians' awareness by reporting this case. Tracheobronchial aspergillosis is presently classified into three distinct clinical patterns: obstructive tracheobronchitis, characterized by thick secretions and mycelial masses with minimal *Aspergillus* mucosal infiltration; ulcerative tracheobronchitis, where fungal hyphae aggressively invade the tracheobronchial mucosa, resulting in ulcerative lesions; and pseudomembranous or necrotizing tracheobronchitis, characterized by a pseudomembrane composed of *Aspergillus* hyphae and necrotic debris [7, 8].

Pseudomembranous *Aspergillus* tracheobronchitis commonly afflicts patients with compromised immune function, including those undergoing prolonged corticosteroid therapy, organ transplant recipients, individuals with malignancies, AIDS patients, or those experiencing malnutrition post‐major surgery. Nonetheless, instances also sporadically manifest in patients with mild immunosuppression stemming from conditions like diabetes and COPD [2, 9, 10]. Furthermore, the patient was diagnosed with influenza A after admission. Studies have shown that influenza has emerged as an independent risk factor for invasive aspergillosis. Recent investigations have underscored the pivotal role of airway epithelium damage and disruption of the tracheal epithelial barrier in the development of invasive *Aspergillus* tracheobronchitis among influenza patients [11]. Additionally, the influenza virus may disrupt host immune regulation by impeding the NADP oxidase (NOX) complex, thereby compromising tracheal defenses against *Aspergillus* [12]. In the context of this case involving a patient with type 2 diabetes, susceptibility to *Aspergillus* in diabetic individuals may be linked to intra‐ and extracellular levels of reactive oxygen species (ROS). Studies have indicated that heightened intracellular ROS levels correlate closely with neutrophil and lung tissue damage, while reduced extracellular ROS levels diminish the bactericidal capacity of neutrophils [13]. Moreover, hyperglycemia could impede the expression of HIF‐1α, an anti‐inflammatory hypoxia‐inducible factor‐1α, thereby disrupting the NLRP3/IL‐1β signaling pathway and influencing T lymphocyte differentiation, thereby compromising the body's anti‐inflammatory response against *Aspergillus* [14]. The patient's blood sugar was not well controlled and was always in a state of high blood sugar before admission. Therefore, the recent hyperglycemia may also be an important factor leading to his pseudomembranous *Aspergillus* tracheobronchitis.

Regarding therapeutic strategies, voriconazole stands as the principal antifungal agent for managing *Aspergillus* tracheobronchitis. In this case, the patient began the treatment of voriconazole after diagnosis of pseudomembranous *Aspergillus* tracheobronchitis, and the trachea and bronchial recovered significantly after 2 months of treatment. This shows the importance of voriconazole in the first‐line treatment of pseudomembranous *Aspergillus* tracheobronchitis. However, there is currently no standardized therapeutic protocol for pseudomembranous Aspergillus tracheobronchitis. In clinical practice, most pseudomembranous Aspergillus tracheobronchitis cases are empirically managed according to treatment guidelines for IPA. Voriconazole dosing and treatment duration similarly align with IPA therapeutic standards [15, 16, 17, 18]. In cases where voriconazole is contraindicated, alternatives such as liposomal amphotericin B and other triazole antifungals can be considered [19]. Noteworthy expert opinions suggest that intratracheal administration of voriconazole may yield beneficial effects in managing *Aspergillus* tracheobronchitis in patients unable to receive intravenous formulations [7, 19, 20].

In conclusion, given the elusive nature of pseudomembranous *Aspergillus* tracheobronchitis in diagnosis, maintaining a vigilant bronchoscopic approach becomes paramount. This becomes especially crucial in patients presenting with recurrent pyrexia and hemoptysis in the absence of clear‐cut pulmonary radiological findings, necessitating heightened surveillance for potential tracheobronchial abnormalities. Currently, the preferred therapeutic approach for *Aspergillus* tracheobronchitis involves the use of voriconazole, administered either intravenously or orally. Additionally, the potential benefits of intratracheal voriconazole instillation in managing *Aspergillus* tracheobronchitis warrant consideration.

## Author Contributions

**Xiaolong Li:** validation, writing – original draft, writing – review and editing. **Xin Wang:** writing – review and editing. **Shuhao Xu:** data curation, writing – review and editing. **Yinhe Feng:** writing – review and editing. **Yuanyuan Liu:** writing – review and editing. **Chunfang Zeng:** writing – review and editing.

## Ethics Statement

This case was approved; The Ethics Committee of the People's Hospital of Deyang City (2024‐04‐059‐K01).

## Consent

The patient gave written informed consent for publication of medical information and images.

## Conflicts of Interest

The authors declare no conflicts of interest.

## Data Availability

Data sharing not applicable to this article as no datasets were generated or analyzed during the current study.
